# Traceable stiffness calibration of colloidal AFM probes for biomechanical measurements

**DOI:** 10.1038/s41598-026-38158-7

**Published:** 2026-02-05

**Authors:** Zhi Li, Valeriya Cherkasova, Sai Gao, Thomas Fröhlich, Uwe Brand

**Affiliations:** 1 Physikalisch-Technische Bundesanstalt, Bundesallee 100, 38116 Braunschweig, Germany; 2 Institute of Process Measurement and Sensor Technology, Technische Universität Ilmenau, 98684 Ilmenau, Germany

**Keywords:** Cantilever stiffness calibration, Atomic force microscopy (AFM), Bio-AFM, biomechanical measurement, AFM colloidal probes tip-surface interaction, Quasi-static stiffness measurement, Nanofriction, Nano-sliding contact, Nano-force metrology, Engineering, Materials science, Nanoscience and technology, Physics

## Abstract

The accurate calibration of bending stiffness of colloidal atomic force microscopy (AFM) probes is essential for reliable nanomechanical measurements, especially when large micro-spheres are used in biological applications. This study investigates the influence of frictional contact between an AFM spherical tip and the load button on stiffness measurements obtained via bending tests and proposes a new analytical model to account for this effect. Finite element simulations of frictional sliding contact between colloidal spheres and load button were conducted to validate the proposed model. A proof-of-principle experimental setup was developed to traceably acquire force-deflection curves of several typical colloidal AFM probes, and results showed good agreement (within 1.5 % deviation) with a validated stiffness calibration system. Experimental data for large-sphere colloidal probes confirmed the presence of a transition phase in the unloading curve due to frictional contact and demonstrated that accurate stiffness results can be obtained when friction is properly considered. Additionally, friction coefficients for four tip-surface material combinations were experimentally determined, providing broadly relevant data that can be effectively applied in AFM nanomechanics, especially in investigations of tip-sample interactions.

## Introduction

Atomic force microscopy (AFM)-based nanomechanical measurements play a crucial role in various scientific and industrial fields, particularly in the development of advanced soft materials^1^ including biomaterials^2–5^. In recent years, bio-AFM nanomechanical phenotyping has emerged as a powerful tool for early disease detection and diagnosis^6,7^, thanks to its exceptional spatial resolution down to the sub-nanometer scale and its ultra-high force sensitivity in the piconewton ($10^{-12}$ N) range. As a result, it has been widely adopted in cell and tissue biology, as well as biomedicine, to address critical questions related to human health and disease^8–10^ and future pandemics^11^. However, achieving reliable and quantitative measurements with bio-AFM requires careful attention to critical instrumentation issues^12–14^. Among these, one of the key challenges is the traceable and accurate calibration of AFM cantilever bending stiffness^15–21^.

Established calibration methods for conventional AFM probes, such as rectangular cantilevers with relatively sharp tips, have been well standardized^22^. One widely adopted traceable quasi-static approach involves using a traceably calibrated micro-force measurement facility^23^, e.g. a precision compensation balance with a force resolution down to nanoNewton (nN, $10^{-9}$ N)^21^ to measure the reaction force of an AFM probe under incremental deflection. This method enables high sensitivity and low measurement uncertainty, even for cantilevers with nominal stiffness as low as 0.01 N/m^24^.

In the meantime, bio-AFM applications often utilize probes with spherical tips, commonly referred to as colloidal probes, with radii ranging from a few microns to tens of microns^25,26^. These are particularly suited for quantitative mechanical characterization of soft biomaterials such as biological tissues. However, applying the standard traceable method to calibrate the bending stiffness of colloidal probes presents significant challenges, primarily due to adhesion and frictional interactions between the probe tip and the load button surface. In addition, it is worthwhile to mention that there exist non-contact indirect stiffness calibration approaches for large colloidal probes on basis of the thermal noise method^27^ or optical tweezers^28^. However, such measurement results need generally to be carefully verified to ensure traceability.

To address this, we propose in this manuscript an analytical model for extracting the effective bending stiffness of colloidal AFM probes from their force–deflection curves. Finite element analysis (FEA) is employed to experimentally investigate the influence of tip-surface friction during bending and to validate the analytical model. A proof-of-principle experimental setup has been developed to investigate the bending stiffness of representative colloidal probes, including custom-fabricated designs. The experimental results support the validity of this proposed model.

## Materials and methods

### Determination of the bending stiffness of AFM cantilevers using a micro-force measurement facility

**Fig. 1:**
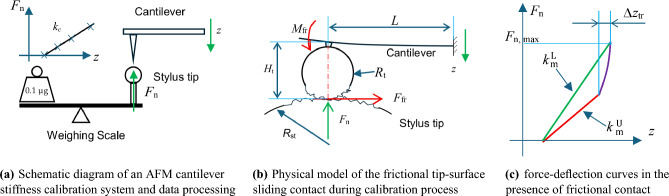
Determination of the quasi-static bending stiffness of colloidal AFM probes using a traceably calibrated compensation balance based microforce measurement facility.

The quasi-static method for the determination of the bending stiffness $k_c$ of an AFM cantilever is illustrated in Fig. 1(a): An AFM cantilever under calibration is positioned by a nano-positioning system (not shown) and engaged onto the load button mounted on a weighing scale, e.g. a compensation balance. For simplicity, in the schematic drawing, the AFM cantilever beam is horizontally mounted. After engagement, the cantilever is further moved downwards, and the reaction force $F_n$ of the cantilever beam under deflection will be directly acquired by the weighing scale. In case that, (1) the AFM tip should be smooth, have relatively small tip radius, be well centered with respect to the load button, and (2) the cantilever bending deflection $\Delta z$ is small, the force-increasing (or loading) and force-decreasing (unloading) curves should overlap well, and demonstrate excellent linearity. The bending stiffness of the cantilever $k_c$ can be finally determined by evaluation of the slope of the loading and unloading ($F - \Delta z$) curves.

On the contrary, typical colloidal AFM probes usually have glued spheres with relatively large tip radius and poor surface quality for material testing, leading to evident frictional (sliding) contact with the load button for bending stiffness measurement, as illustrated in Fig. 1(b). And, in practice, the tip radius $R_{st}$ of the load button is usually far larger than the AFM tip radius $R_t$, i.e. $R_{st}>> R_t$. And quite often, instead of a rounded load button, a flat punch will be utilized for AFM cantilever bending measurements.

### Theoretical analysis of the frictional tip-surface sliding contact for stiffness evaluation

Under the applied normal force $F_n$, the friction force $F_{fr}$ between the AFM spherical tip and the load button can be estimated by

1 $$\begin{aligned} F_{fr} = \mu \cdot F_n, \end{aligned}$$

where $\mu$ denotes the Coulomb’s coefficient of friction between the colloidal tip and the load button. Notably, the sign before $\mu$ depends on the direction of tip sliding. Specially, during unloading $F_{fr} = - \mu \cdot F_n$.

Under the steady-state frictional sliding contact between the AFM spherical tip and the load button, the vertical deflection $\Delta z$ of colloidal probe due to the normal contact force $F_n$ and the friction force $F_{fr}$ can be deduced as follows:

2 $$\begin{aligned} \Delta z = \Delta z_{F_n} - \Delta z _{M_{fr}}, \end{aligned}$$

where $M_{fr} = \mu F_n \cdot H_t$ is the equivalent moment on the cantilever beam caused by friction force $F_{fr}$, $H_t$ the equivalent tip height of the AFM spherical tip, and

3 $$\begin{aligned} & \Delta z_{F_n} = \frac{F_n \cdot L^3}{3 \cdot E I}, \end{aligned}$$

4 $$\begin{aligned} & \Delta z_{M_{fr}} = \frac{M_{fr} \cdot L^2}{2 \cdot E I} = \frac{\mu \cdot F_n \cdot H_t \cdot L^2}{2 \cdot E I} , \end{aligned}$$

where *L* is the equivalent cantilever length at the contact point, *E* the elastic modulus of the cantilever beam, and *I* is the second moment of area of the beam’s cross section^29^.

Finally, we have the total deflection of the cantilever at the contact point

5 $$\begin{aligned} \Delta z = \left[ 1 - \frac{3}{2} \mu \frac{H_t}{L} \right] \frac{F_n \cdot L^3}{3 E I} , \end{aligned}$$

Given the cantilever stiffness $k_c = 3 \cdot EI/L^3$, the measured stiffness $k_m$ from the $F -\Delta z$ curves can be estimated as follows:

6 $$\begin{aligned} \frac{1}{k_m} = \left[ 1 - \frac{3}{2} \mu \frac{H_t }{L} \right] \cdot \frac{1}{k_c}. \end{aligned}$$

It can be seen from Eq. 6 that the measured cantilever stiffness $k_m$ suffers from the influence of tip-surface contact friction. The influence will become stronger, when the ratio of tip height to cantilever length $H_t/L$ increases. In addition, from Eq. 6 the measured stiffness from the steady-state part of the loading curve $k_m^L$ and the measured unloading stiffness $k_m^U$ can be deduced as follows:

6a $$\begin{aligned} \begin{aligned} k_m^L = [1-\frac{3}{2} \mu \frac{H_t}{L} ]^{-1} \cdot k_c \\ k_m^U = [1+\frac{3}{2} \mu \frac{H_t}{L} ]^{-1} \cdot k_c \end{aligned} \end{aligned}$$

Obviously, the measured $k_m^L$ is usually larger than $k_m^U$, as shown in Fig. 1(c). The cantilever stiffness $k_c$ can be therefore conveniently deduced from Eq. 6a as

7 $$\begin{aligned} k_c = 2 \left[ \frac{1}{k_m^L} + \frac{1}{k_m^U} \right] ^{-1} = 2 \frac{ {k_m^L} \cdot k_m^U}{ k_m^L + k_m^U }. \end{aligned}$$

From Eq. 6a we obtain

7a $$\begin{aligned} 3 \mu \frac{H_t}{L} \cdot \frac{1}{k_c} = \frac{1}{k_m^L} - \frac{1}{k_m^U} = \frac{k_m^L - k_m^U }{ {k_m^L} \cdot k_m^U }. \end{aligned}$$

Combing Eq. 7a with Eq. 7, the friction coefficient $\mu$ can be further derived as follows:

8 $$\begin{aligned} \mu = \frac{1}{3} \frac{L}{H_t} \cdot k_c \cdot \frac{k_m^L - k_m^U}{ k_m^L \cdot k_m^U} = \frac{2}{3} \frac{L}{H_t} \frac{k_m^L - k_m^U}{ k_m^L + k_m^U} . \end{aligned}$$

It is also very interesting to note that at the beginning of the unloading curve, there should exist a transition phase, since the frictional force will change its direction. From Eq. (4), the transition range $\Delta z_{tr}$ can be estimated as follows:

9 $$\begin{aligned} \Delta z_{tr} = \frac{3}{2} \mu \frac{H_t}{L} \frac{F_{n, max}}{ k_c} \approx \frac{3}{2} \mu \frac{H_t}{L} \Delta z_{max}, \end{aligned}$$

where $\Delta z_{\textrm{max}}$ is the maximum deflection of the cantilever beam during measurement.

To evaluate the unloading stiffness $k_m^U$ precisely, it is therefore recommended that only measurement data out of the transition phase should be utilized for data evaluation.

### Finite element analysis of frictional tip-surface sliding contact during bending measurement

**Fig. 2:**
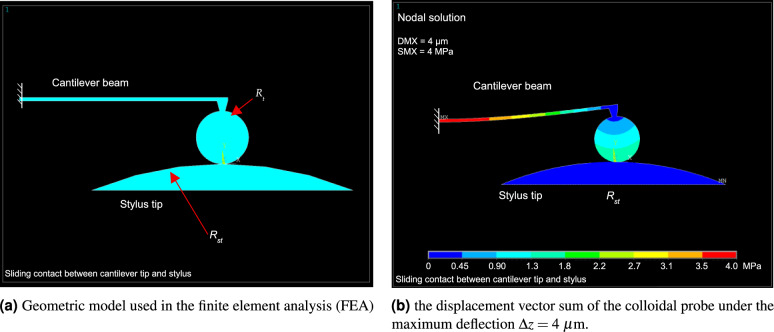
FEA-based numerical investigation of the tip-surface interaction during the stiffness calibration process.

To validate the theoretical analysis in Eqs. (1)-(9), the frictional contact between a colloidal sphere and the load button during the stiffness measurement process has been numerically investigated with the help of finite element (FE) analysis. The geometrical model used in FE simulation is illustrated in Fig. 2(a). The fundamental parameters of the cantilever beam, glued sphere, and the load button are listed in Table 1. And the ratio $H_t/L$ amounts to about 1/3.

**Table 1:** Parameter table of the colloidal AFM probe and the load button used in the finite element analysis.

	**Dimensions**			**Materials**	Young’s modulus, GPa	Poisson’s ratio
Colloidal AFM probe*	Beam	Length *L*, $\mu$m	150	Si	169	0.28
		Thickness *T*, $\mu$m	2.5			
		Width *w*, $\mu$m	30			
	Tip	Height $H_t$, $\mu$m	50	Glass	63	0.17
		Radius $R_t$, $\mu$m	20			
Load button		Radius $R_{ST}$, $\mu$m	250	Ruby	400	0.3

*Cantilever beam dimensions come from the datasheet of AFM probe HQ: XSC11 series, $\upmu$Masch.

For simplicity, a two-dimensional FE analysis was carried out using Ansys Mechanical APDL version 19.2 (Ansys Inc., USA). During the loading (and unloading) process, the load button is fixed, only the left end of the cantilever is moved downwards (and upwards). Typical displacement vector sum of the colloid probe and the load button under maximum deflection is illustrated in Fig. 2(b).

**Fig. 3:**
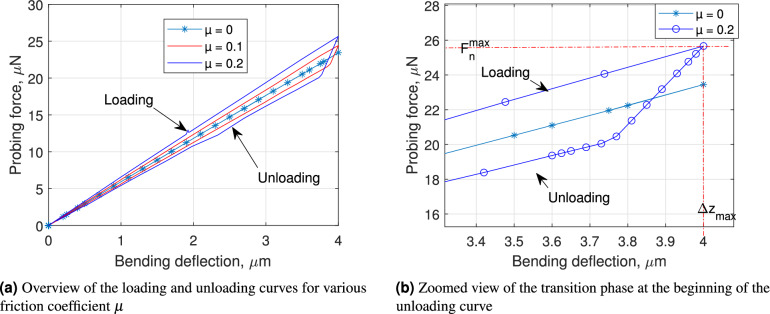
Numerical investigation of the force-deflection curves for AFM cantilever stiffness measurement under different frictional tip-surface sliding.

The numerical simulation results are presented in Fig. 3. As shown in Fig. 3(a), when the friction coefficient $\mu = 0$, the loading and unloading curves overlapped quite well, displaying excellent linearity across the entire deflection range of 4 $\mu$m. As $\upmu$ increasing, the loading stiffness $k_m^L$ becomes noticeably greater than the unloading stiffness $k_m^U$. In particular for $\mu = 0.2$, the transition range calculated using Eq. (9) is $\Delta z_{\textrm{tr}} = 0.40$ $\mu$m, which agrees well with the numerical analysis illustrated in Fig. 3(b).

## Results

To experimentally validate the data analysis model proposed above, a proof-of-principle experimental setup has been developed, with which home-made and commercial colloidal AFM probes have been measured.

### A proof-of-principle micro-force measurement facility for traceable measurement of the normal stiffness of colloidal AFM probes with relatively large spheres

**Fig. 4:**
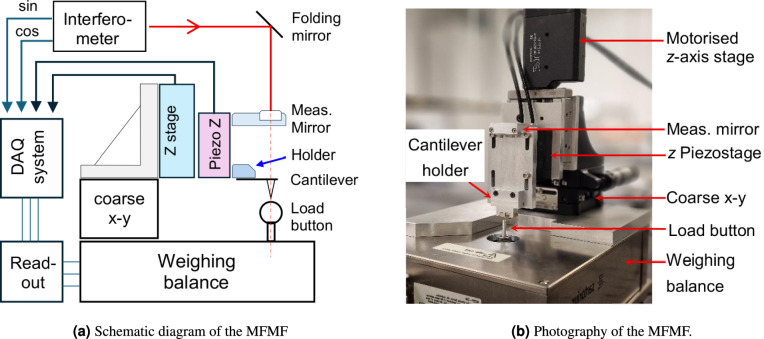
Home-developed micro-force measurement facility (MFMF) for traceable calibration of the bending stiffness of AFM probes.

The home-developed micro-force measurement facility (MFMF) consists of a traceably calibrated compensation balance (Sartorius AG, WZ2P-CW, linearity $\le$ 20 nN, standard deviation $\le$ 20 nN) for reading the reaction force of a cantilever under calibration, a nanopositioning system (P-620, Physik Instrumente GmbH) for moving the cantilever, and a three-axis coarse positioning system, as illustrated in Fig. 4(a).

A custom-built data acquisition system with a LabView-based user interface has been developed to drive the motorized z-axis stage for coarse positioning of the cantilever under calibration. Fine engagement of the cantilever with the load button on the balance, along with the subsequent bending measurement, is achieved by actuating the z-axis piezo stage. To enable in-situ calibration of the combined z-positioning system, including both the motorized z-axis stage and the nanopositioning piezo-stage, a home-built laser interferometer (not depicted in Fig. 4(b)) is employed.

### Typical measurements of classical AFM probes

The MFMF has been firstly employed to measure the normal stiffness of classical AFM probes with a diamond flat punch load button (20 $\mu$m in diameter, fabricated by Synton AG, Switzerland). Using the motorized z-stage, the cantilever tip was positioned approximately 15 $\mu$m above the top surface of the load button. The z-piezo stage was then used to move the AFM probe incrementally toward the load button until the maximum displacement $\Delta z_{\textrm{max}}$ was reached. Each step included a typical waiting time of 5 seconds, corresponding to five times the balance response time, to ensure reliable readings. The actual position of the AFM probe and the corresponding balance output were recorded simultaneously. All measurements were typically performed under controlled environmental conditions, with a temperature of $21 \pm 0.3^\circ$ C and a relative humidity of $38 \pm 4 \%$. The experimental setup was installed on a stable granite table without air damping. Alignment between the AFM probe and the load button was achieved with the help of a home-built auxiliary microscope, resulting in a misalignment below 5 $\mu$m.

**Fig. 5:**
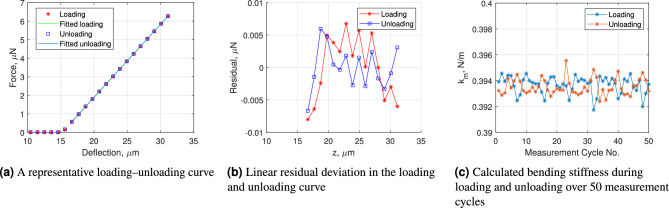
Typical measurement results for determining the normal stiffness of a contact-mode AFM cantilever with a rounded tip (SD-R30-CONT, NanoSensors™).

Figure 5(a) shows one of the force-deflection curves obtained by the MFMF for a contact-mode AFM cantilever with rounded tip (SD-R30-CONT, NanoSensors^™^, $R_t$ = 30 nm, *L* = 450 $\mu$m and $H_t$ = 15 $\mu$m). The maximum cantilever deflection $\Delta z_{\textrm{max}}$ reaches approximately 16 $\mu$m during the measurement, and the total duration of one measurement cycle amounts to 3.5 minutes.

According to Eq. (4), the ratio $H_t/L \approx 0.033$ is relatively small, indicating that the effect of frictional contact between the AFM tip and the load button on stiffness measurements is minimal and can be considered negligible. This is well supported by Fig. 5(a), where the loading and unloading curves overlap closely over a cantilever bending range of 16 $\mu$m. As shown in Fig. 5(b), the residual linear deviation of the loading and unloading curves within the evaluation region for stiffness calculation is less than 10 nN. Figure 5(c) further demonstrates excellent repeatability of the stiffness measurements, with a variation of less than $0.2 \%$ across 50 measurement cycles (approximately 3 hours in total). The measured stiffness of this cantilever is $0.3936 \pm 4 \%$ (a detailed uncertainty analysis is available in^21,24^).

For comparison, the bending stiffness of the same cantilever has also been measured using a traceable force-displacement measurement device developed at TU Ilmenau, employing both electromagnetic (EMFC) and electrostatic force (ESFC) compensation principles^24,30^. These methods yielded results of $k_m^{\textrm{EMFC}} = 0.3930 \pm 0.0046$ N/m and $k_m^{\textrm{ESFC}} = 0.3968 \pm 0.0022$ N/m, respectively. The stiffness measured using the proof-of-principle MFMF clearly agrees well with those obtained by the validated measurement system at TU Ilmenau.

### Typical stiffness measurements of colloidal AFM probes

**Fig. 6:**
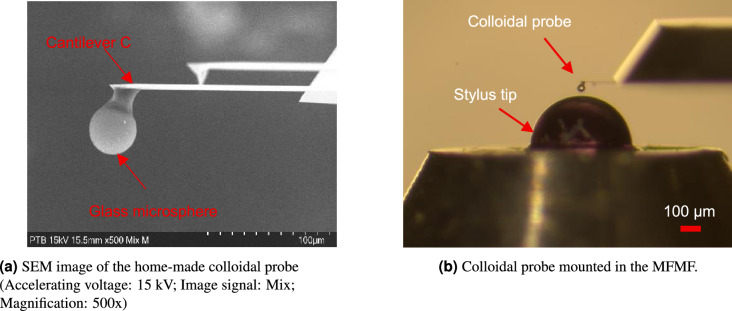
Traceable calibration of the bending stiffness of a home-made colloidal AFM probe.

A home-made colloidal probe was fabricated by attaching a Soda Lime glass microsphere ($30 \pm 20$ $\mu$m in diameter, Goodfellow GmbH) to a HQ AFM cantilever (HQ: XSC11, Cantilever C, $\mu$Masch Europe), as illustrated in Fig. 6(a). The three-dimensional topography of the contact surface of the microsphere was characterized using a laser scanning confocal microscope (LEXT OLS4100, Olympus), yielding a surface roughness of $S_a = 47$ nm in the central contact region. This colloidal probe has an equivalent cantilever length of $L = 150$ $\mu$m, and a total tip height of approximately 50 $\mu$m.

A home-made load button with a ruby half-ball (Edmund Optics GmbH, 500 $\mu$m in diameter) as the testing stylus has been used to measure the bending stiffness of the colloidal probe, as shown in Fig. 6(b). Topography measurement of the ruby half-ball by the aforementioned confocal microscope reveals that the top surface of the load button has a roughness of $S_a = 13$ nm.

**Fig. 7:**
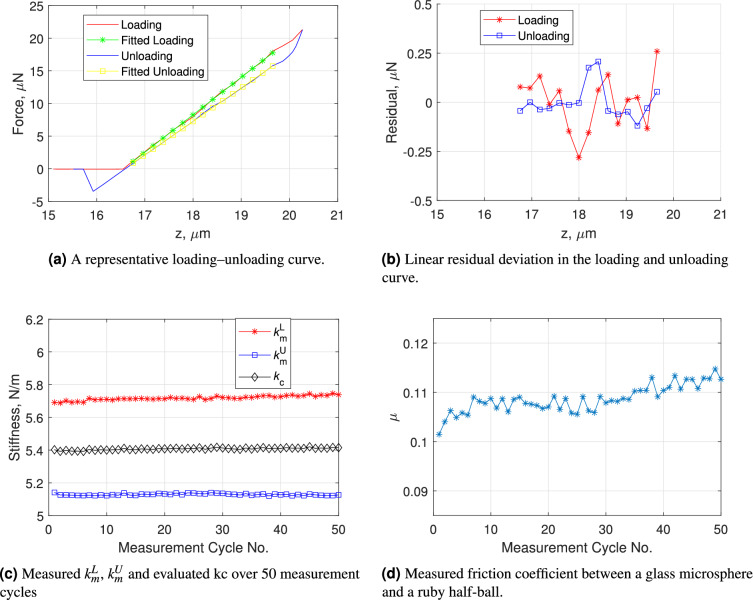
Typical measurement results for determining the normal stiffness of a home-fabricated colloidal probe.

Figure 7(a) presents a representative force-deflection ($F - \Delta z$) curve acquired using the MFMF for this home-fabricated colloidal probe. Notably, two key features are observed: (1) the loading $F - \Delta z$ curve exhibits a different slope compared to the unloading curve, and (2) a distinct transition phase is evident at the onset of unloading.

By performing linear fitting in the respective linear regions of the loading and unloading curves, as indicated in Fig. 7(a), the measured loading stiffness $k_m^L$ and unloading stiffness $k_m^U$ are quantitatively determined. Figure 7(b) details the residual deviation from linearity within these regions. Interestingly, the magnitude of the residual deviation is approximately $\pm 0.25$ $\mu$N, which is nearly 20 times larger than the deviations observed in Fig. 5(b). This larger deviation is attributed to the relatively high surface roughness of the microsphere ($S_a = 47~\mathrm{nm}$ and $S_q = 75~\mathrm{nm}$ ) attached to the cantilever. Figure 7(c) summarizes the measured values of $k_m^L$, $k_m^U$, along with the calculated cantilever stiffness $k_c$ derived using Eq. (7). Additionally, Figure 7(d) reports the measured friction coefficient $\mu$ between the glass microsphere and the ruby ball load button. During the sequence of contact bending measurements, slight variation in $\mu$ were observed, resulting in the minor fluctuations in $k_m^L$ and $k_m^U$. Despite this, the cantilever stiffness remains stable, yielding $k_c = 5.41 \pm 0.01$ (repeatability, 1$\sigma$) N/m, demonstrating excellent consistency throughout the measurement series.

**Fig. 8:**
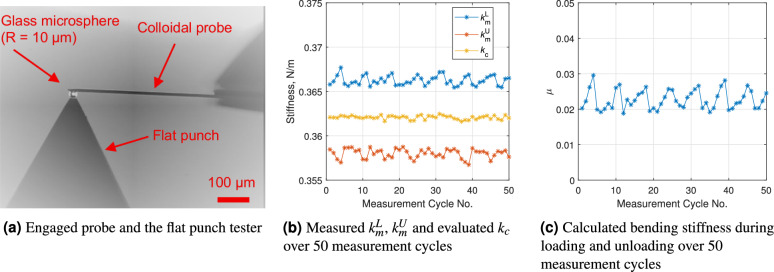
Typical measurement results for determining the normal stiffness of a contact-mode colloidal AFM probe (CP-CONT-BSG, sQube).

A commercial colloidal AFM probe (CP-CONT-BSG, sQube, Borosillicate Glass sphere, 20 $\mu$m in diameter, cantilever length $L = 450$ $\mu$m, nominal stiffness 0.36 N/m) has also been calibrated using the MFMF with a diamond flat punch, as shown in Fig. 8(a). With the same measurement procedure and data analysis method detailed above, the measured loading, unloading stiffness and deduced stiffness of this commercial colloidal probe is depicted in Fig. 8(b). The measured friction coefficient between a glass microsphere and a diamond flat punch is illustrated in Fig. 8(c). We can conclude that the stiffness of this commercial colloidal cantilever $k_c = 0.362 \pm 4 \%$ N/m and $\mu _{\mathrm{glass-diamond}} = 0.023 \pm 0.003$.

Finally, a commercial colliodal AFM probe with glued PMMA microsphere (CP-CONT-PM-E, sQube, 15 $\mu\mathrm{m}$ in diameter) has also been measured with the above mentioned MFMF and procedure. For convenience, the measurement results of the cantilevers investigated in this manuscript are summarized in Table 2.

**Table 2:** Summary of the experimental results reported in this manuscript.

**AFM probes**		**Reference cantilever**	**Colloidal probes**		
		SD-R30-CONT, NanoSensors^TM^	HQ: XSC11, $\upmu$Masch (glued microsphere, R = 20 $\upmu$m)	CP-CONT-BSG, (R = 10 $\upmu$m), sQube	CP-CONT-PM, (R = 7.5 $\upmu$m), sQube
**Beam Dimensions, **$\upmu$ **m**	*L*	450	150	450	450
	$H_t$	15	50	20	15
**Stiffness** ± **4%, N/m**	$k_m^{L}$	0.394	5.718	0.366	0.487
	$k_m^{U}$	0.394	5.129	0.360	0.470
	$k_c$	0.394	5.408	0.362	0.478
**Friction coefficient,** $\mu$		$\mu _{\mathrm{Si-Diamond}}$ = 0.00	$\mu _{\mathrm{Glass-Ruby}}$ = 0.11	$\mu _{\mathrm{Glass-Diamond}}$ = 0.02	$\mu _{\mathrm{{PMMA-Diamond}} }$ = 0.04
**Stiffness by TU Ilmenau**, N/m	$k_m^{EMFC}$	0.3930 ± 0.0046	na	0.3570 ± 0.0055	na
	$k_m^{ESFC}$	0.3968 ± 0.0022	na	0.3568 ± 0.0016	na

## Conclusion

In this manuscript, we have systematically analyzed and modeled the influence of frictional contact between AFM tips and load buttons on stiffness measurement results obtained using the quasi-static bending-based calibration method. A new data evaluation model has been developed for the quantitative determination of the bending stiffness of colloidal AFM probes, particularly those equipped with large micro-spheres. The proposed analytical model has been validated through finite element simulations of representative frictional sliding contacts between a colloidal probe and a load button.

A proof-of-principle experimental setup was constructed to traceably acquire force-deflection ($F - \Delta z$) curves from several representative AFM probes. Initial results indicate that, for stiffness calibration of standard AFM probes, the home-built micro-force measurement facility yields results that are in excellent agreement with those obtained from the validated force-displacement measurement device, with deviations of less than $1.5 \%$.

Force-deflection measurements on colloidal AFM probes with relatively large spheres confirm the key predictions of the analytical model: (1) a distinct transition phase appears at the beginning of the unloading curve due to frictional contact effects, and (2) accurate stiffness results can still be achieved when these frictional contributions are properly accounted for. Additionally, friction coefficients for several typical tip-surface combinations, including silicon-diamond (nanoscale), glass-diamond (microscale), PMMA-diamond (microscale), and glass-ruby (submillimeter scale), were experimentally determined. These coefficients are of broad relevance and can be utilized in future research and development efforts in AFM nanomechanics, particularly in studies of tip-sample interactions.

It should be noted that the present theoretical analysis does not account for the influence of geometrical form errors of the colloidal probe spheres, which may contribute to measurement uncertainty. Future work will address this limitation through quantitative modeling. Moreover, the current analysis does not include the effects of adhesion forces $F_{\text {JKR/DMT}}$^31^, which may become significant when calibrating the stiffness of ultra-soft cantilevers. In the regime where $F_{\text {JKR/DMT}} \gg F_n$, the friction force in Eq. (1) becomes $F_{fr} = \mu F_{\text {JKR/DMT}}$, and the transition range $\Delta z_{tr}$ can be estimated as

10 $$\begin{aligned} \Delta z_{tr} = \frac{3}{2} \mu \frac{H_t}{L} \cdot \frac{F_{\text {JKR/DMT}}}{k_c} \equiv \text {Constant}. \end{aligned}$$

Meaningful measurements can only be obtained when the total deflection $\Delta z_{\text {max}} \gg \Delta z_{tr}$. Further development of the analytical model to encompass these effects will be pursued to enable accurate characterization of ultra-soft AFM colloidal probes with large spheres, particularly for biomechanical measurement applications.

In addition, stiffness calibration in this manuscript was performed using quasi-static measurements; therefore, only quasi-static tip–button interactions were considered. In contrast, for fast stiffness calibration approaches-such as those employing high-speed piezostages and high-speed force sensors, including reference springs-the friction force between the AFM tip and the load button becomes dependent on sliding velocity and exhibits generally nonlinear behavior^32,33^. Under these conditions, the analytical model proposed here is no longer valid and requires further extension.

Finally, it is worth noting that most commercial AFMs operate with a tilted cantilever. However, the analytical model proposed in this manuscript is formulated for stiffness measurements of untilted AFM cantilevers. For in-situ stiffness calibration of tilted colloidal AFM probes, for example, using an in-situ nanoforce sensor, an extension of the proposed analytical model will be required to accommodate this commonly used configuration.

## Supplementary Information

Supplementary Information 1.

Supplementary Information 2.

## Data Availability

The data obtained and used in this contribution can be provided by the corresponding author upon request.
