# Supplementary material for: Traceable stiffness calibration of colloidal AFM probes for biomechanical measurements
Source: Sci Rep. 2026 Feb 5;16:5243. doi: 10.1038/s41598-026-38158-7 (PMC12880979; doi:10.1038/s41598-026-38158-7)
Supplement: Supplementary file 1 — Supplementary Information 1. [file 41598_2026_38158_MOESM1_ESM.pdf]

Supplementary Material

Finite Element Modeling of Frictional Tip–Surface Sliding in Colloidal AFM Cantilever Stiffness Measurements

Zhi Li[1,*], Valeriya Cherkasova[2] , Sai Gao[1] , Thomas Fröhlich [2] , and Uwe Brand[1]

[1]Physikalisch-Technische Bundesanstalt, Bundesallee 100, 38116 Braunschweig, Germany

[2]Institute of Process Measurement and Sensor Technology, Technische Universität Ilmenau, 98684 Ilmenau, Germany

*zhi.li@ptb.de

To quantitatively evaluate the effect of frictional contact between a colloidal sphere and a load button on cantilever stiffness measurements, finite element (FE) analysis was performed using the commercial software ANSYS (version 19.2).

Although a fully 3D model would provide a more accurate representation of the frictional contact between the AFM colloidal probe and the load button, our focus is on the numerical assessment of the bending stiffness of a long, slender cantilever. Under conditions of small cantilever deflections and proper alignment between the colloid and the load button, a plane-stress approximation for the cantilever is reasonable. Accordingly, the interaction between the colloidal AFM probe (cantilever plus microsphere) and the load button can be effectively captured using a simplified 2D model.

Section 1 details the FE analysis of frictional sliding contact between a glass colloid and a ruby half-sphere load button. Section 2 presents the FE analysis of frictional sliding contact between a glass colloid and a diamond flat punch load button, corresponding to the measurements shown in Figure 8 of the main manuscript.

1. FE analysis of the sliding contact between a colloidal probe with glued glass micro-sphere and a ball-shaped load button.

1.1 Modeling/Preprocessing

Geometrical Model

As illustrated in Figure S-1, the silicon cantilever (A1) and its colloidal tip (A2, a glass microsphere) are glued together. The load button (A3) is modeled as a segment of a circular plate, since the deformation of the ruby ball is negligible in the simulation.

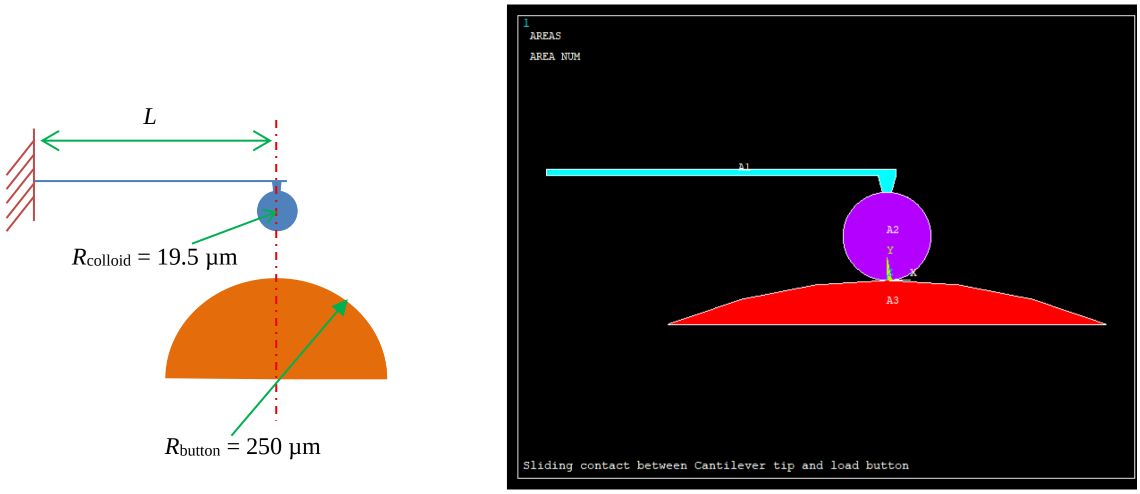

(**a**) Schematic of the tip-button contact system (**b**) CAD model

Figure S-1 CAD model for sphere-sphere contact

Table S-1 Geometrical model

Numbering	*Object*	*Material*
A1	AFM cantilever beam	Silicon
A2	Microsphere (glued)	Glass
A3	Load button	Ruby

Meshing

All objects were meshed using triangular elements, as shown in Figure S-2. It is well known that the element size defined in the simulation plays an important role in determining the accuracy of the results. Assuming a cantilever beam thickness of *T*, the element sizes (ES) for each object were chosen to balance accuracy and computational efficiency as follows:

- Element size for the cantilever beam: ES_b = T/N,
- Element size for the microsphere: $ES_s = 4 \times ES_b$,
- Element size for the load button: $ES_t = 4 \times ES_b$,

where N is an integer.

In addition, the contact pair between the colloid and the load button has been further refined to increase the simulation accuracy, as shown in Figure S-2. For frictional contact, Coulomb's law of friction is applied directly, such that the tangential friction force F_t is proportional to the normal contact force F_n, i.e., $F_t = \mu F_n$, where μ denotes the coefficient of friction.

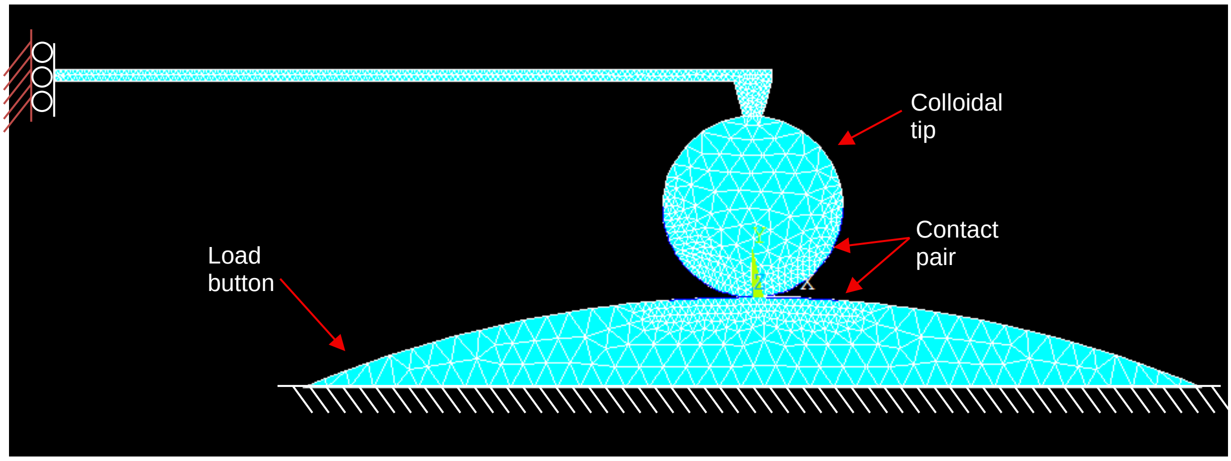

Figure S-2 Meshing and constraints

Material and Property Information

Once meshing is completed, material properties (e.g. Young's Modulus and Poisson's ratio) are assigned to the elements.

Table S-2 Material properties used in FEA

**Object**	**Material**	**Material property**	
		Young's modulus E, GPa	Poisson's ratio, *v*
A1	Silicon [1]	169	0.28
A2	Glass [2]	63	0.17

A3	Ruby [3]	400	0.3

1.2 Solution

Loads, Constraints and Solver Information

As shown in Figure S-2, in the 2D FE simulation, the bottom surface of the load button (Ruby ball) is fixed (i.e. UX = UY = 0), and the left end of the AFM cantilever is moved vertically, i.e. UX = 0. The colloid of the AFM probe has initially contact with the top surface of the load button, i.e. no gapping.

Although the cantilever bending deflection Δz over the whole measurement procedure is relatively small, with respect to its beam length L, a static analysis will be performed with automatic time stepping and large-deflection/-strain effects included, to ensure a better simulation accuracy.

For the simulation of the three-point surface-to-surface contact, the contact algorithm "*Pure Lagrange multiplier on contact normal and tangent*" [4] was selected. This algorithm enforces zero penetration when the contact is closed and ensures zero slip under sticking contact conditions. It does not require the specification of contact stiffness parameters, including the normal contact stiffness factor (*FKN*) and the tangential contact stiffness factor (*FKT*).

1.3 Simulation results

1.3.1 Mesh Convergence Study

Mesh convergence in finite element analysis (FEA) is widely regarded as the benchmark for assessing simulation accuracy. For the cantilever bending analysis shown in Figures S-1 and S-2, it is expected reducing the element size, such that it is significantly smaller than the cantilever thickness T, enhances numerical accuracy.

Under the condition of μ = 0 and $UY_{\mathrm{cantilever}}$ = 4 μm, the relationship between the element size ES_{b} and the calculated stiffness k_{c} has been numerically investigated. As shown in Figure S-3 the numerically calculated cantilever stiffness k_{c} converges to an approximately constant value when the element size ES_b is less than one quarter of the cantilever thickness, i.e., $T/ES_b \geq 4$. Consequently, an element size of $ES_b = T/4$ is adopted for all subsequent numerical simulations.

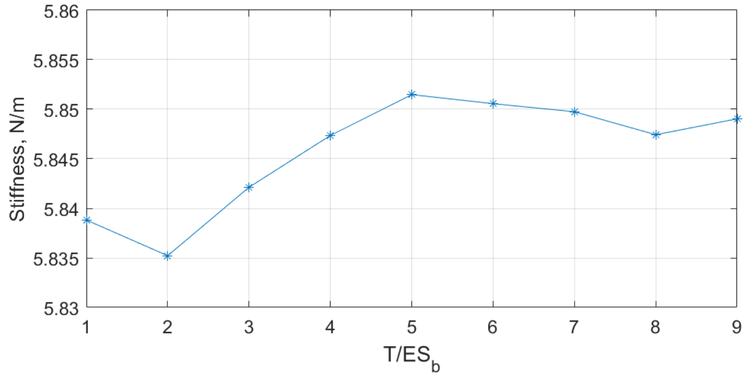

Figure S-3 Mesh Convergence Study: Numerical assessment of the effect of element refinement on the calculated bending stiffness.

It is noteworthy that for a rectangular cantilever with a width w (= 30 μm), thickness T (= 2.5 μm), and length L (= 150 μm), the analytical bending stiffness is given by

$$k_c = \frac{EwT^3}{4L^3} = 5.868 \text{ N/m.} \qquad (1)$$

This indicates that the numerical result is in excellent agreement with the analytical calculation.

2. FE analysis of the sliding contact between a colloidal probe with glued glass micro-sphere and a diamond flat punch

2.1 Preprocessing

In case that a diamond flat punch is used as the load button, as shown in Figure S-4(a), a new CAD model is created in Figure S-4(b). Similar to the preprocessing in subsection 1.1, the CAD model is meshed and illustrated in Figure S-4 (c) and (d).

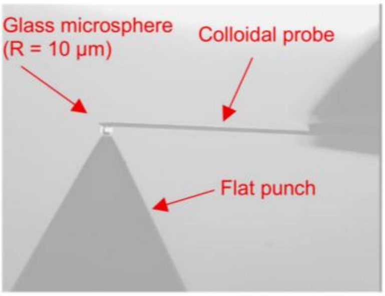

(**a**) Stiffness measurement of a colloidal probe with a flat punch as the load button

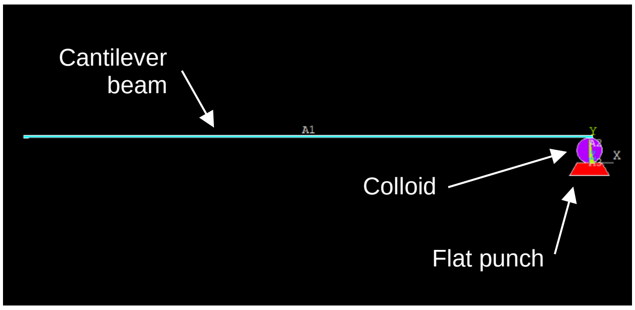

(b) CAD model

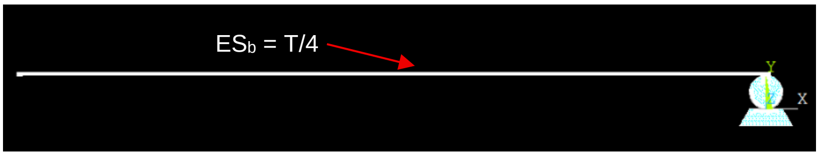

(**c**) Meshed FEM model

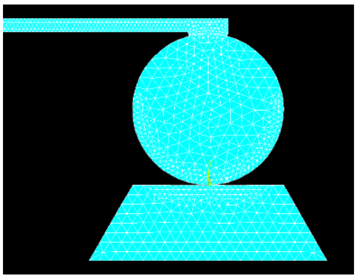

(d) Zoomed FEM model for simulating tip-button contact

Figure S-4 Finite element modeling for the sliding contact between a colloid and a flat punch load button

2.2 Numerical results

For a silicon cantilever with length L = 450 µm, thickness T = 2.5 µm and width w = 50 µm, its analytical stiffness calculated from Eq. (1) is k_c = 0.36 N/m.

To numerically investigate the sliding contact between the colloidal probe and the flat punch load button, the material properties listed in Table S-3 were employed in the finite element analysis.

Figure S-5(a) shows the cantilever deformation under a maximum deflection of UY = 18 µm during bending measurements. Figure S-5(b) and S-5(c) present the numerically obtained force-deflection curves for different friction coefficients for the measurement system depicted in Figure S-4(a).

Table S-3 Material properties used in FEA for the sliding contact shown in Figure S-4.

Object	Material	Material property	
		Young's modulus E, GPa	Poisson's ratio, *v*
A1	Silicon [1]	169	0.28
A2	Glass [2]	63	0.17

A3	Diamond [5-6]	1141	0.07

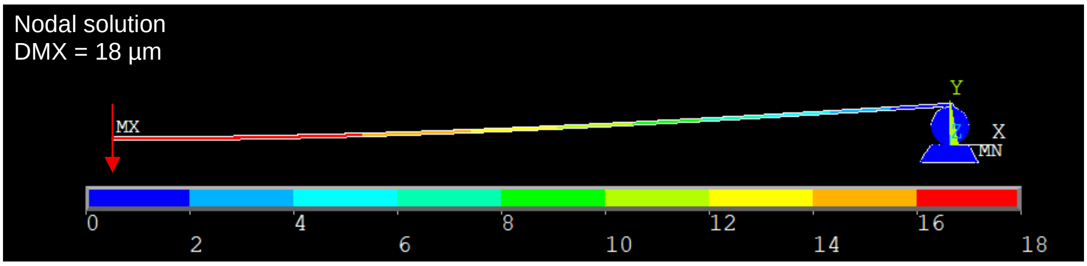

(a) Displacement vector sum of the colloidal AFM probe under maximum deflection

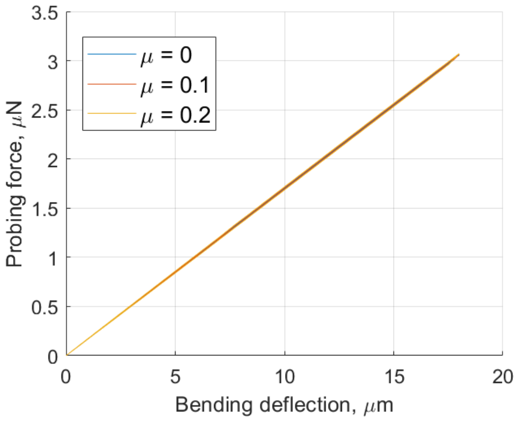

(**b**) Overview of the loading and unloading curves for various friction coefficient μ

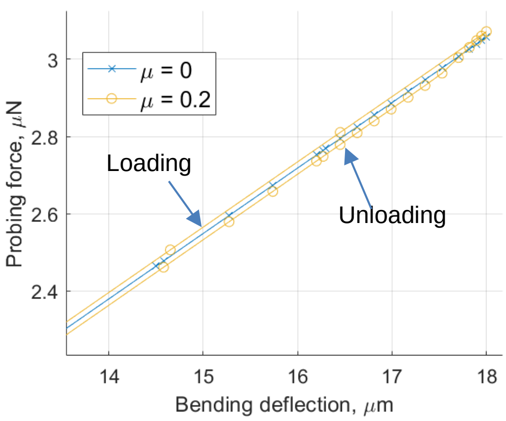

(**c**) Zoomed view of the transition phase at the beginning of the unloading curve

Figure S-5 Numerical results

The simulation results indicate that the effect of frictional contact on cantilever bending measurements can be neglected when the cantilever length *L* is much larger than the colloid radius $R_{colloid}$, in agreement with the predictions of our analytical analysis.

References

[1] De Laat, M.L.C.; Pérez Garza, H.H.; Ghatkesar, M.K. In situ Stiffness Adjustment of AFM Probes by Two Orders of Magnitude. Sensors 2016, 16, 523. https://doi.org/10.3390/s16040523

[2] A. Chorfa , M. A. Madjoubi , M. Hamidouche , N. Bouras , J. Rubio , F. Rubio, "Glass hardness and elastic modulus determination by nanoindentation using displacement and energy methods", Ceramics-Silikáty 54(2010) 225-234

[3] J. Nohava, J. Mencík, “A contribution to understanding of low-load spherical indentationdcomparison of tests on polymers and fused silica”, J. Mater. Res. 27 (2011) 239-244.

[4] ANSYS Mechanical APDL Contact Technology Guide. Chapter 3: "Surface-to-Surface Contact (Pair-Based)". Ansys, Inc.; Canonsburg, PA, USA: 2025. pp. 15-143.

[5] Field, J.E.; Telling, R.H. The Young Modulus and Poisson Ratio of Diamond; PCS Cavendish Laboratory: Cambridge, UK, 1999.

[6] L. De Fazio, S. Syngellakis, R.J.K. Wood, F.M. Fugiuele, G. Sciume, "Nanoindentation of CVD diamond: comparison of an FE model with analytical and experimental data", Diam Relat Mater, 10 (3–7) (2001), pp. 765-769
