# Supplementary material for: Traceable stiffness calibration of colloidal AFM probes for biomechanical measurements
Source: Sci Rep. 2026 Feb 5;16:5243. doi: 10.1038/s41598-026-38158-7 (PMC12880979; doi:10.1038/s41598-026-38158-7)
Supplement: Supplementary file 2 — Supplementary Information 2. [file 41598_2026_38158_MOESM2_ESM.pdf]

Supplementary Material

Surface characterisation of colloid and load buttons using a commercial confocal microscope

To investigate the surface characteristics of the colloids and load buttons, including surface roughness, a commercial confocal microscope (Olympus LEXT OLS4100) was used to quantitatively measure the topography of their contact surfaces. This instrument employs a short illumination wavelength of 405 nm, providing a lateral resolution of down to 200 nm when using an objective with NA = 0.95, and an axial resolution of up to 10 nm.

Measurements were performed on:

- the flat-punch load button shown in Figure 8(a),
- the ruby half-ball shown in Figure 6(b), and
- the colloid shown in Figure 6(a).

It is worth noting that this well-calibrated instrument has been extensively used for the quantitative topographical characterization of a wide range of micro-objects, including Rockwell indenter tips (R = 200 µm) [1]. The glass colloid in Figure 6(a) has a diameter of approximately 40 µm, the ruby half-ball has a diameter of 500 µm, and the flat punch has an in-plane diameter of approximately 20 µm. Owing to their dimensions, these specimens can be rapidly, conveniently, and quantitatively characterized using this confocal microscope.

1. Surface characteristics of the flat punch load button in Figure 8(a)

The top surface of the flat punch was characterized using the confocal microscope equipped with a high–numerical aperture objective (NA = 0.95, 100×). Figure S-1(a) presents the corresponding intensity image, while the measured surface topography is shown in Figure S-1(b).

After levelling, a detailed view of the central region of the flat-punch surface is provided in Figure S-1(c). The peak-to-valley (PV) value within the central area (R ≤ 5 µm) was determined to be 10.5 nm. A line profile extracted at y = 15 µm is shown in Figure S-1(d), from which an arithmetic mean roughness of Ra = 0.4 nm was obtained using the Gwyddion software.

Further surface analysis of the central region yielded an areal roughness Sa = 1.6 nm and a root-mean-square roughness Sq = 1.9 nm.

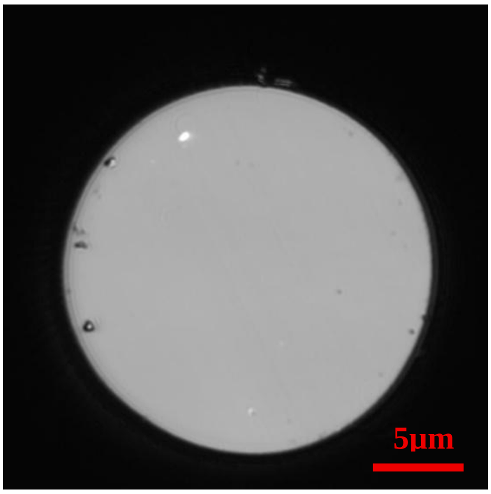

(**a**) Intensity image

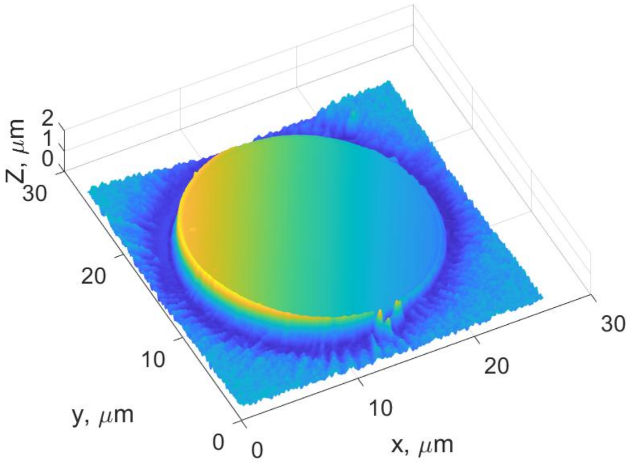

(b) 3D topography of the top surface of the flat punch

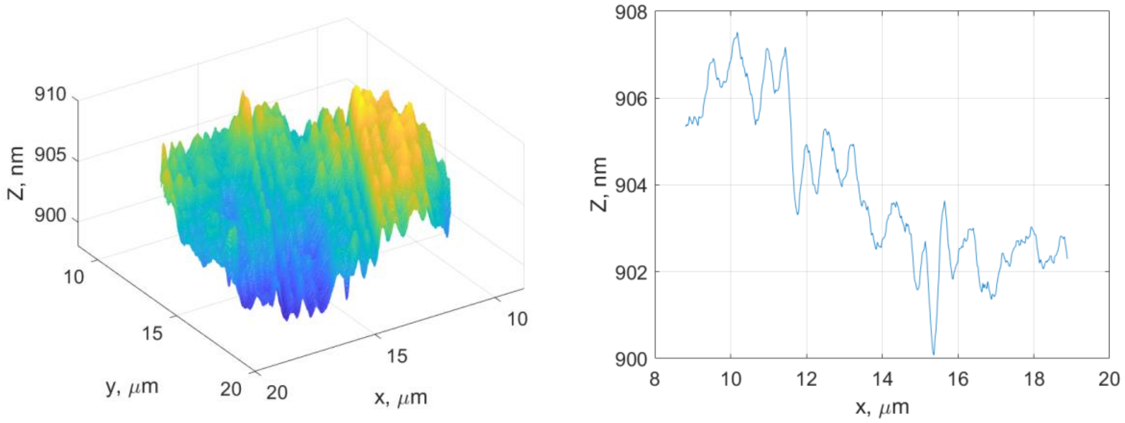

(**c**) Levelled topography of the flat punch's central part

(d) Measured line profile at y = 15 µm.

Figure S-1 Evaluation of the surface characteristics of a flat punch load button using a confocal microscope

2. Surface characteristics of the ruby half-ball shown in Figure 6(b)

Using the same confocal microscope described in Section 1 and a 50x objective with NA = 0.95, the ruby half-ball was imaged. Figure S-2(a) shows the measured surface topography of the half-ball. A three-dimensional spherical fit was applied to the data, yielding a radius of R_{ruby} = 245.3 µm. The line profile z_{origin} of the half-ball at y = 135 µm is illustrated in Figure S-2(b). For comparison, the corresponding fitted line profile $z_{fitting}$ is also overlaid in the same figure.

The residual profile error, defined as $\Delta Z = z_{origin} - z_{fitting}$ at y = 135 µm is detailed in Figure S-2(c). The residual topography error at the central region of the ruby half-ball (i.e. a diameter of 30 µm) is detailed in Figure S-2(d).

Using the software *Gwyddion*, the surface roughness within this central region was evaluated, yielding Sq = 13.3 nm and Sa = 10.3 nm.

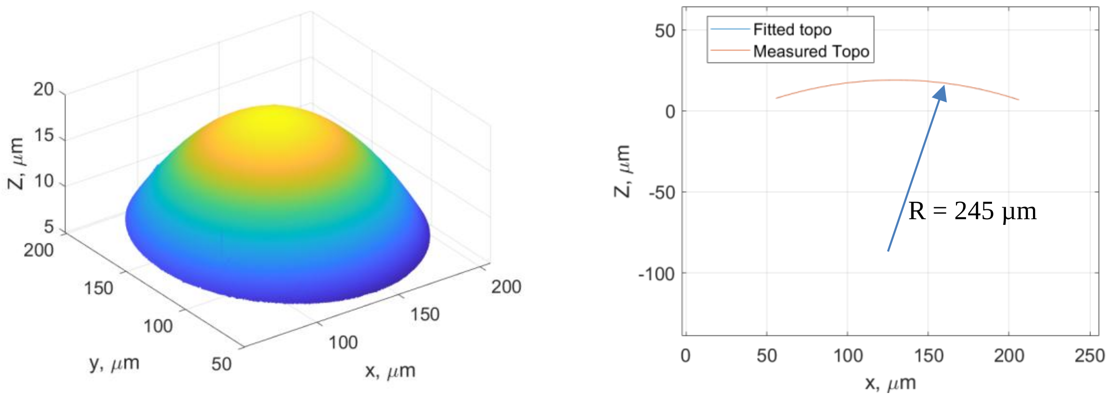

(**a**) 3D topography of the top surface of half-ball

(b) Line profile of the half-ball at y = 135 µm

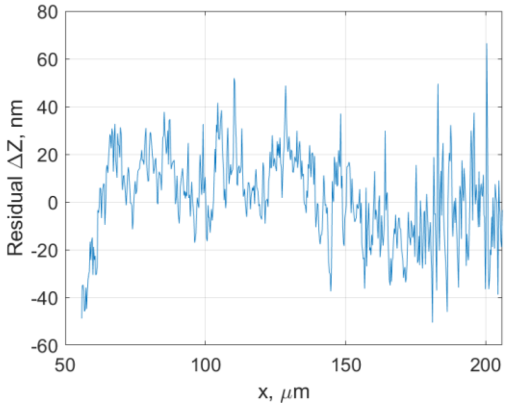

(**c**) Residual line profile error at y = 135 µm

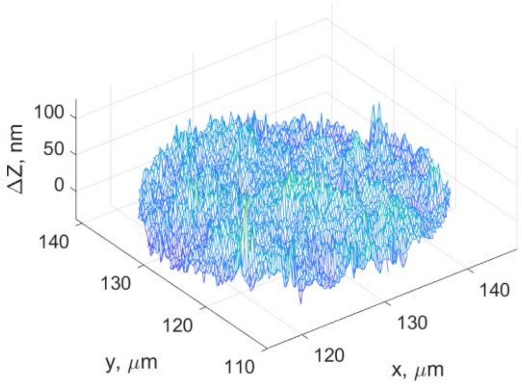

(d) Residual surface topographical error within the central region of the ruby half-ball .

Figure S-2 Evaluation of the surface characteristics of the ruby half-ball using a confocal microscope

3. Surface characteristics of the colloid shown in Figure 6(a)

Using the same confocal microscope and the same objective mentioned in Section 1, the glass colloid shown in Figure 6(a) was imaged. Figures S-3(a) shows the SEM image of the colloid. Figure S-3(b) and S-3(c) illustrate the intensity and the measured topography of the colloid, respectively. A three-dimensional spherical fit was applied to the data matrix in Figure S-3(c), yielding a radius of R_{tip} = 20.4 µm ± 6 nm.

After removal of the underlying spherical form, the residual topography error within the central region of the colloid's top surface (approximately 13 µm x 13 µm) is detailed in Figure S-3(d).

Using the software *Gwyddion*, the surface roughness within this central region was evaluated, yielding Sq = 75 nm and Sa = 47 nm.

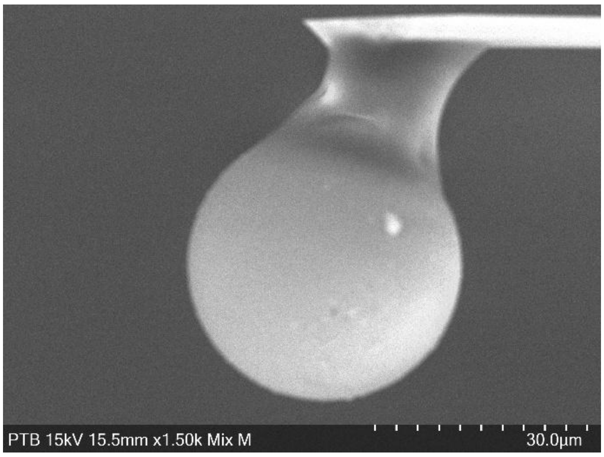

(**a**) SEM image of the glass colloid

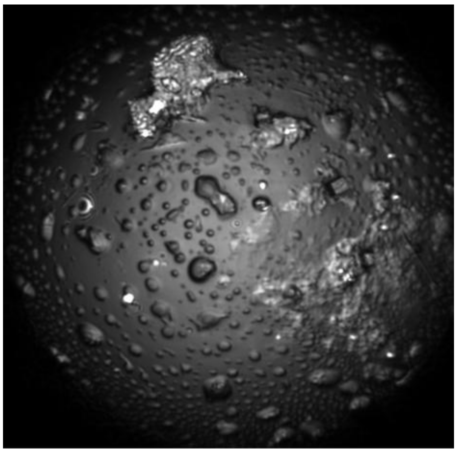

(b) Intensity image of the colloid

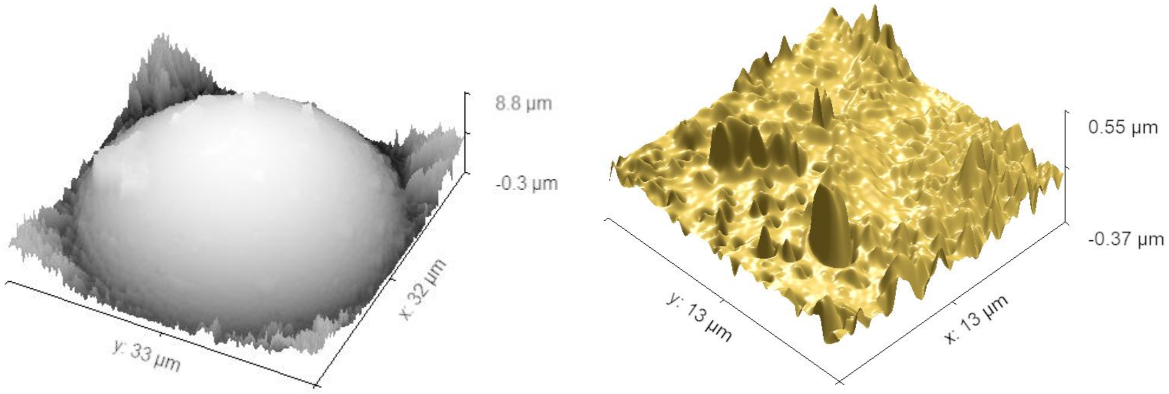

(**c**) 3D topography of the top surface of the colloid **(d)** Residual surface topographical error within the central region

Figure S-3 Evaluation of the surface characteristics of the glass colloid using a confocal microscope

Summary

For reference, the surface characteristics of the three objects analyzed in this manuscript are summarized in Table S-1.

Roughness Parameters*		Sa, nm	Sq, nm
Flat punch		1.6	1.9
Ruby half-ball	R_{ball} = 245.3 µm	10.3	13.3
Glass Colloid	R_{tip} = 20.4 µm	47	75

* Surface roughness parameters were evaluated within the central region of the top surfaces of the objects.

Table S-1 Surface characteristics of the objects measured by the confocal microscope

References

[1] Mutaib Zackaria et al 2024 Calibration of the spherical tip radius of Rockwell hardness diamond indenters using a confocal laser scanning microscope. *Meas. Sci. Technol.* **35** 125017. DOI: 10.1088/1361-6501/ad6f39
